# Lumbar Artery Pseudoaneurysm Following Renal Biopsy

**DOI:** 10.7759/cureus.2634

**Published:** 2018-05-16

**Authors:** Basit Salam, Kumail Khandwala

**Affiliations:** 1 Department of Radiology, The Aga Khan University, Karachi, PAK

**Keywords:** lumbar artery, pseudoaneurysm, coil embolization, renal biopsy

## Abstract

Lumbar artery pseudoaneurysms have previously been described as rare iatrogenic complications following percutaneous interventional procedures involving the flanks. We describe a case of a 71-year-old man who became unstable and dropped 3 grams of hemoglobin within 24 hours following renal biopsy. A post-biopsy hemorrhage was suspected, and a pseudoaneurysm of his second right lumbar (L2) artery was found on computed tomography angiogram (CTA). Successful coil embolization was performed in the right L2 artery. This case discusses the diagnostic and therapeutic challenges of this unusual complication as well as the anatomical and technical factors involved in the embolization of the lumbar arteries.

## Introduction

A percutaneous renal biopsy is routinely performed by nephrologists and interventional radiologists to diagnose various types of renal diseases. It is supposed to be a relatively safe procedure with a low risk of complications, especially with the adjunctive use of image guidance [1]. Vascular complications after renal biopsy are mostly related to the native kidneys. Hematuria or blood in the collecting system, parenchymal or perinephric hematomas, intrarenal pseudoaneurysms, and arteriovenous fistulas are examples of such complications that have been widely reported in previous literature [1-2]. Lumbar artery pseudoaneurysms following a renal biopsy are a particularly rare occurrence. We describe a case of a 71-year-old man who developed a massive retroperitoneal hematoma due to a pseudoaneurysm if his second right (L2) artery following a renal biopsy that was subsequently managed with endovascular coil embolization.

## Case presentation

**Figure 1:**
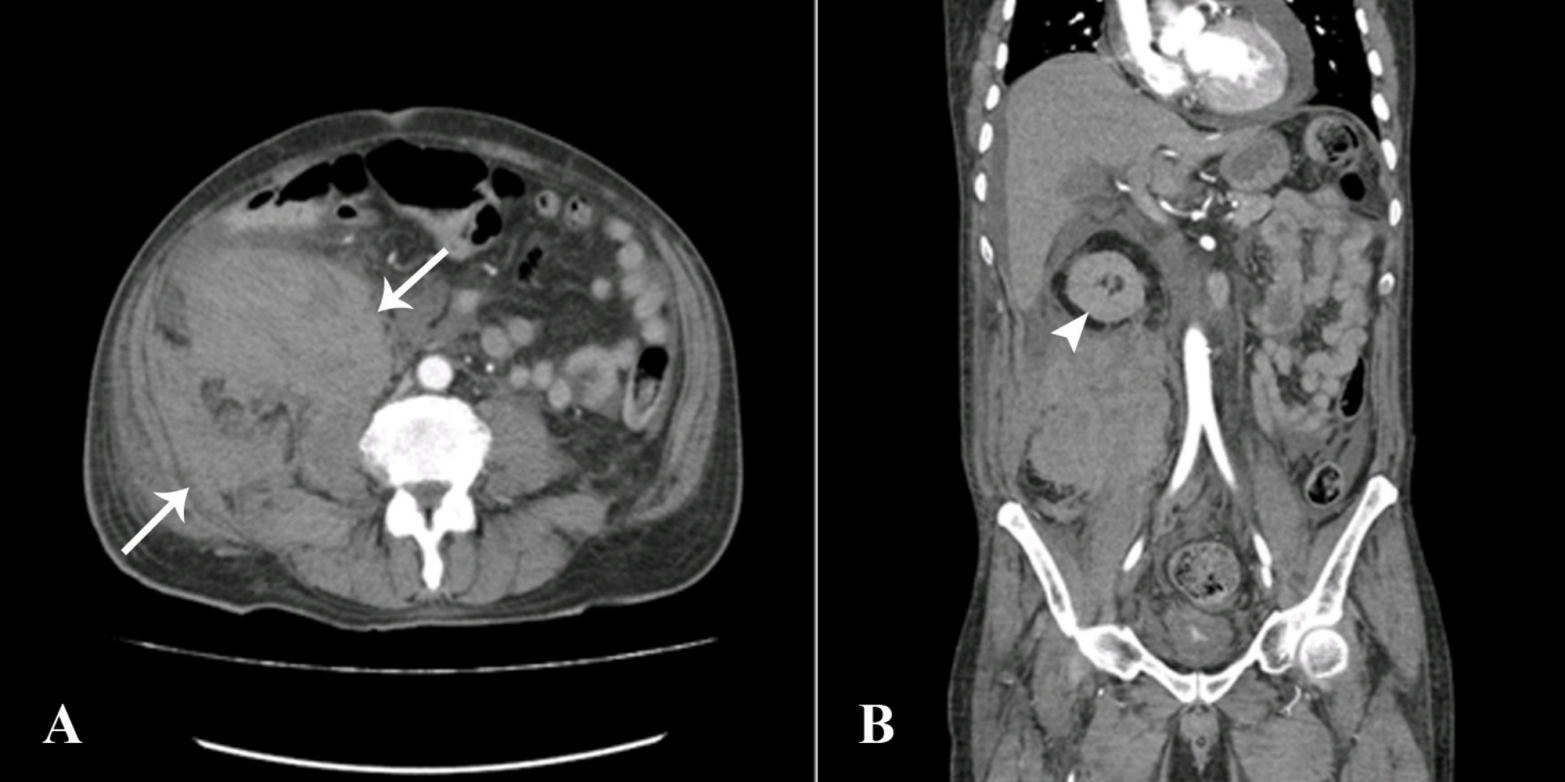
CT abdomen and pelvis angiogram A) Axial section showing a large right-sided retroperitoneal hematoma (arrows) B) Coronal section showing that the hematoma was located separately from the lower pole of the right kidney (arrowhead) CT: computed tomography

A 71-year-old male patient, an ex-smoker, underwent a right-sided renal biopsy for an acute kidney injury and the derangement of renal function (creatinine: 8.1 mg/dL and blood urea nitrogen: 74 mg/dL). The derangement of renal function was believed to be secondary to vasculitis as his peripheral anti-neutrophil cytoplasmic antibodies (P-ANCA) levels were positive and, therefore, crescentic glomerulonephritis was suspected. Post-procedure, the patient became hemodynamically unstable within the first 24 hours and dropped 3 grams of hemoglobin. Hemoglobin/hematocrit before the procedure was 10.7 gm/dL/30.4%. Hemoglobin/hematocrit after the biopsy was 6.5 gm/dL/19.2%. His coagulation profile was normal. The patient had a contrast-enhanced computed tomography (CT) angiogram (CTA) for a suspected post-biopsy hemorrhage, which showed no active contrast extravasation from the native kidneys. Instead, a large retroperitoneal hematoma was seen in the right posterior lumbar and iliac fossa region, which was separate from the lower pole of the right kidney (Figure 1).

**Figure 2:**
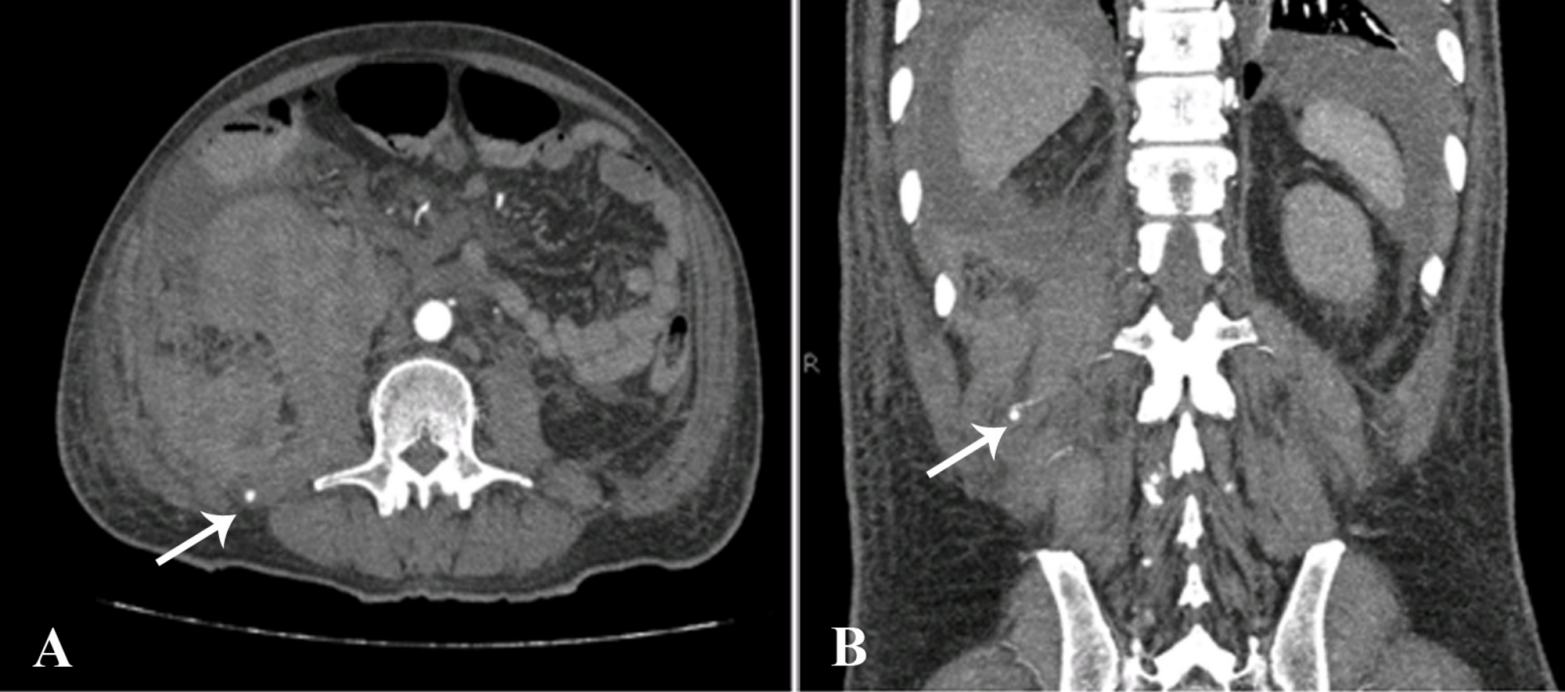
CT abdomen and pelvis angiogram Axial (A) and coronal (B) images from the arterial phase showing a small saccular pseudoaneurysm arising from the right L2 lumbar artery (arrows). CT: computed tomography

The hematoma was measuring 11 cm in craniocaudal dimensions. On the arterial phase, a small saccular pseudoaneurysm measuring 3 mm was seen arising from the right second lumbar artery posterior to this hematoma (Figure 2).

**Figure 3:**
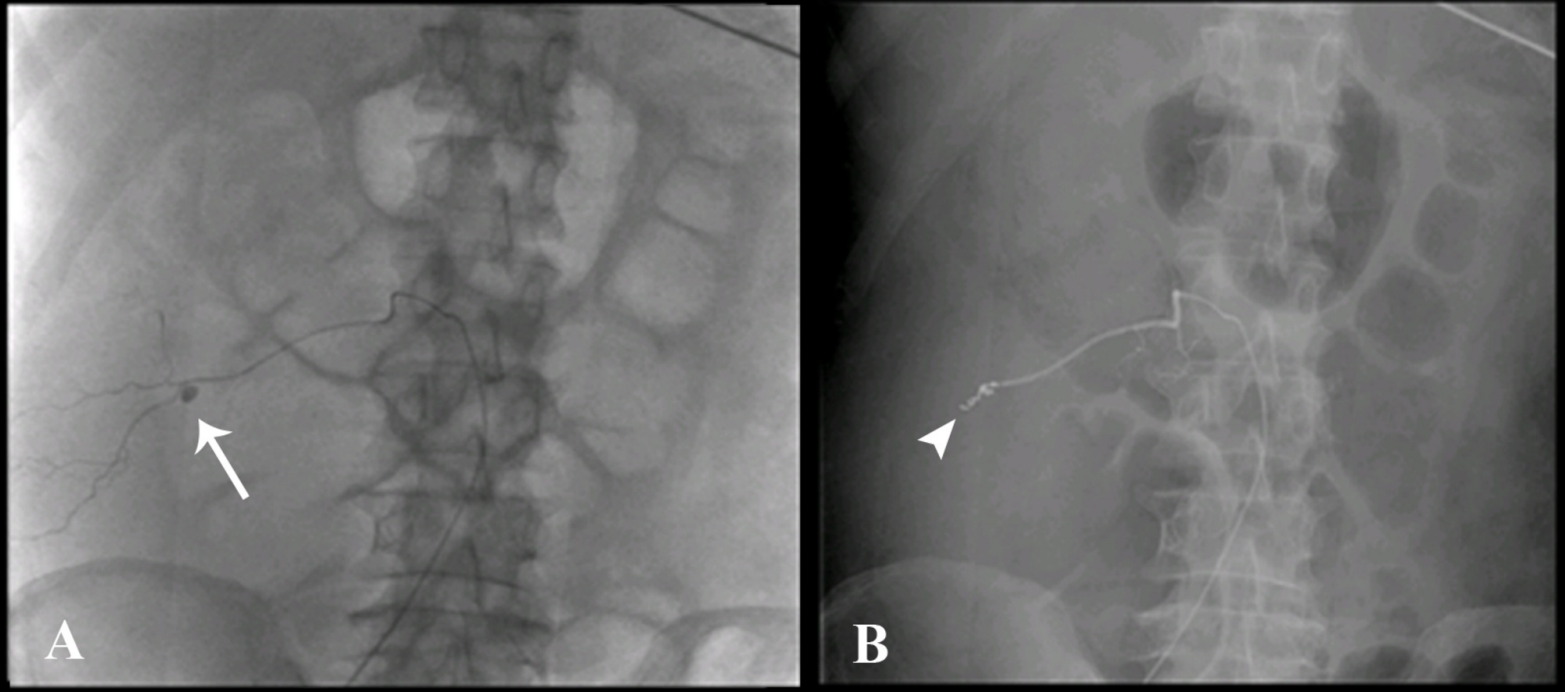
Conventional angiogram A) Angiographic images confirming the pseudoaneurysm in the right L2 lumbar artery (arrow) B) Successful post-coiling of the pseudoaneurysm (arrowhead)

Subsequently, conventional angiography was performed. An initial abdominal aortogram was performed via a right common femoral arterial approach. Catheterization of the second left lumbar artery was performed with a 4 Fr Cobra catheter. Selective catheterization of the branch with the pseudoaneurysm was done with a microcatheter. Subsequently, coil embolization was done with three coils (one distal and two proximal to the pseudoaneurysm) followed by Gelfoam pledget embolization. The final angiogram demonstrated the successful exclusion of the pseudoaneurysm with a preserved flow in the main trunk of the lumbar artery (Figure 3).

A selective renal angiogram was then performed that showed no evidence of renal vascular injury. Post-embolization, the patient had no further episodes of bleeding and was discharged in a stable state.

## Discussion

A percutaneous renal biopsy is a relatively safe technique to perform with the use of imaging guidance. Renal hematomas are the most commonly reported complications, but a study shows that more than 90% of these are self-limited, with only 6% becoming clinically significant and effecting morbidity [1]. However, the first 24 hours remain a critical observation period for post-biopsy related complications. Other complications like arteriovenous fistulas, renal artery pseudoaneurysms, colonic injuries, infections, or pneumothorax are much less frequent, with an incidence of less than 0.1% [2].

A lumbar artery pseudoaneurysm is a rare complication that has been previously described following percutaneous nephrostomy and nephrolithotomy, vertebral biopsy, radical nephrectomy, and post-traumatic avulsion injuries [3-7]. Lumbar and intercostal artery pseudoaneurysms are a particularly unusual occurrence after a renal biopsy, with only a handful of case reports documented in the past [8-10]. The lumbar arteries (L1 to L4) are paired vessels that arise from the posterior aspect of the aorta at the level of transverse processes [5]. They then run posterolaterally along the vertebral bodies and divide into small branches supplying the psoas muscle and to the radicular medullary artery before dividing into anterior and posterior branches medial to the psoas muscle. The anterior branch supplies the quadratus lumborum and sacrospinalis muscle and then gives off branches to the muscles and skin of the flank. The posterior branch supplies the sacrospinalis and skin on the back. Both these branches run in a posterior relation to the kidney and are, therefore, at risk of iatrogenic injury during percutaneous procedures [5-6]. The presence of a posterior pararenal space or a retroperitoneal hemorrhage should, therefore, strongly raise the suspicion of a lumbar artery injury. Despite their small size and retroperitoneal location, these pseudoaneurysms are potentially lethal once they rupture due to the resultant blood loss and shock.

Surgical management is usually not recommended since the site of origin of bleeding may not be easily detected, and operative exploration of the retroperitoneum may lift the tamponade effect of the hematoma and, therefore, potentially increasing the risk of bleeding. Angiography and embolization is a minimally invasive, quick technique that accurately localizes the bleeder and makes immediate therapeutic effect possible. It also avoids the risks involved with general anesthesia and has, therefore, been effectively used in the past for the successful treatment of lumbar artery pseudoaneurysms [3-4,6-8]. In our case, a transcatheter embolization successfully eliminated the pseudoaneurysm as well.

However, special attention needs to be paid to technical and anatomical factors for arterial embolization involving the lumbar arteries. The anterior radicular artery (artery of Adamkiewicz) may originate anywhere from the sixth intercostal to the second lumbar artery and, therefore, must be avoided to prevent spinal-cord-related complications or injuries. Hence, liquid agents for embolization in this anatomical location are generally not advisable. Coil embolization should be performed distally first and then proximal to the pseudoaneurysm to prevent the pseudoaneurysm from refilling via collateral supply. Coil embolization may be reinforced with gel foam to achieve adequate hemostasis. If the tip of the catheter is close to the origin of the radicular medullary artery, a forceful injection should be avoided to prevent spinal cord injury [6]. No neurological complications have occurred following the embolization of the lumbar artery pseudoaneurysm in our patient.

## Conclusions

In summary, vascular complications related to a renal biopsy mostly involve the native kidneys themselves and are usually self-limited. A lumbar artery pseudoaneurysm following percutaneous renal procedures is an unusual complication and may present as a major diagnostic and therapeutic challenge. Coil embolization supplemented with gel foam pledgets is a safe and effective method of controlling the bleeding and eliminating the pseudoaneurysm. However, special attention must be given to the anatomical and technical factors that are involved, especially avoiding the spinal branches to minimize spinal-cord-related complications and injuries. It is important to promptly recognize and treat this type of complication to prevent morbidity and mortality after these relatively common and safe interventional procedures.
